# Prevalence of pain and pharmacological pain treatment among old people in nursing homes in 2007 and 2013

**DOI:** 10.1007/s00228-017-2384-2

**Published:** 2017-12-20

**Authors:** Eva-Stina Hemmingsson, Maria Gustafsson, Ulf Isaksson, Stig Karlsson, Yngve Gustafson, Per-Olof Sandman, Hugo Lövheim

**Affiliations:** 1 Department of Community Medicine and Rehabilitation, Geriatric Medicine, Umeå University, SE-901 87 Umeå, Sweden; 2 Department of Pharmacology and Clinical Neuroscience, Division of Clinical Pharmacology, Umeå University, SE-901 87 Umeå, Sweden; 3 Department of Nursing, Umeå University, SE-901 87 Umeå, Sweden; 4 Arctic Research Centre at Umeå University, SE-901 87 Umeå, Sweden; 5 Department of Neurobiology, Care Sciences and Society, Division of Nursing, Karolinska Institutet, SE-171 77 Stockholm, Sweden; 6 Department of Health Sciences, University of Technology, Luleå, Sweden

**Keywords:** Pain, Elderly, Nursing home, Analgesic drugs, Dementia

## Abstract

**Purpose:**

Many elderly people living in nursing homes experience pain and take analgesic medication. The aim of this study was to analyze the prevalence of pain and pharmacological pain treatment among people living in nursing homes in Sweden, in two large, comparable, samples from 2007 to 2013.

**Methods:**

Cross-sectional surveys were performed in 2007 and 2013, including all residents in nursing homes in the county of Västerbotten, Sweden. A total of 4933 residents (2814 and 2119 respectively) with a mean age of 84.6 and 85.0 years participated. Of these, 71.1 and 72.4% respectively were cognitively impaired. The survey was completed by the staff members who knew the residents best.

**Results:**

The prescription of opioids became significantly more common while the use of tramadol decreased significantly. The staff reported that 63.4% in 2007 and 62.3% in 2013 had experienced pain. Of those in pain, 20.2% in 2007 and 16.8% in 2013 received no treatment and 73.4 and 75.0% respectively of those with pain, but no pharmacological treatment, were incorrectly described by the staff as being treated for pain.

**Conclusions:**

There has been a change in the pharmacological analgesic treatment between 2007 and 2013 with less prescribing of tramadol and a greater proportion taking opioids. Nevertheless, undertreatment of pain still occurs and in many cases, staff members believed that the residents were prescribed analgesic treatment when this was not the case.

## Introduction

With rising age, there is an increased risk of contracting chronic and life-limiting, painful medical conditions [1]. The most common pain sites in older people are the back, leg/knee or hip, and other joints [2]. Pain has been associated with reduced physical and psychological functioning [3, 4] and in interviews, half of the elderly patients admitted for palliative care ranked elimination of pain as their primary need [5].

In people with dementia, pain is common and frequently under-treated [6], and adequate pain evaluation and treatment in people with dementia has been shown to have positive effects [7]. For example, several studies have shown that pain treatment might reduce behavioral and psychological symptoms of dementia [8, 9].

Elderly individuals represent a vulnerable group who are more sensitive to adverse drug effects [10]. According to the American Geriatrics Society, appropriateness of drug treatment lies not only in the choice of drugs but also the choice of appropriate dosage and co-medications [11]. There is also general agreement that a multimodal approach involving both pharmacologic and non-pharmacological treatment (e.g., physical activity, physiotherapy) is a good way of managing pain [12].

In the county of Västerbotten in northern Sweden, recurrent surveys of those living in nursing homes have been undertaken since 1975. Lövheim et al. reported that 56.7% of the elderly living in nursing homes in 2000 was judged to experience pain and a significant proportion of those were untreated [13]. In Sweden, drug treatment among the elderly has been in focus in recent years, and in this context it is of interest to study trends over time in analgesic treatment and pain among elderly in nursing homes.

The aim of this study was to examine the prevalence of pain and changes in pharmacological pain treatment among people living in nursing homes in Sweden, in two large, comparable samples from 2007 to 2013.

## Methods

### Materials

This study is based on two cross-sectional questionnaire surveys completed in 2007 and 2013 in the county of Västerbotten in the northern part of Sweden. The two surveys used the same methodology and the same questionnaire and included all those resident in nursing homes for old people, including specialized care units for people with dementia. A total of 3578 people in 2007 and 3210 in 2013 were included in the investigated population. The response rates in 2007 and in 2013 were 3070 (85.8%) and 2262 (70.5%) persons respectively. In 2007, but not in 2013, people in geriatric and psychogeriatric hospital wards were included in the survey. Therefore, people in hospital wards in 2007 (99 persons) were excluded from the analyses, leaving 2971 people living in nursing homes in 2007 and 2262 persons in 2013. Further, 151 people in 2007 and 127 people in 2013 younger than 65 or for whom no age were registered were excluded. In addition, 6 people in 2007 and 16 people in 2013 for whom no sex were registered were excluded, leaving a final sample of 2814 persons in 2007 and 2119 in 2013. The basic characteristics of the samples are presented in Table 1.

**Table 1:** Characteristics of residents

	2007	2013	*p* value
Cases *n*	2814	2119	
Women *n* (%)	1924 (68.4)	1435 (67.7)	0.627
Mean age ± SD	84.6 ± 6.8	85.0 ± 7.0	0.073
Cognitive score (0–27) mean ± SD	16.2 ± 8.9	16.5 ± 8.4	0.235
Cognitive impairment *n* (%)	1966/2764 (71.1)	1503/2077 (72.4)	0.345
ADL score (4–24) mean ± SD	15.5 ± 6.3	15.6 ± 6.2	0.504
Walks independently *n* (%)	1324/2787 (47.5)	1195/2053 (58.2)	< 0.001
Normal speech, can make themselves understood	2062/2739 (75.2)	1575/1934 (81.4)	< 0.001
Number of regular drugs^a^ ± SD	7.6 ± 3.5	7.7 ± 3.6	0.471

*ADL* activities of daily living, *SD* standard deviation

^a^Among people with complete drug lists, *n* = 2766 (2007), *n* = 1887 (2013)

### Procedures

The study forms were sent to the settings and the staff received written instructions about how to complete the individual assessment form based on observations during one week. The staff who knew the resident best were asked to complete that person’s form. Members of the research team could be contacted by telephone to answer questions if needed. After the form was completed, it was returned to the researchers. In 2007, the assessments were completed by nursing assistants (24.7%), registered practical nurses (66.3%), registered nurses (2.7%), and staff with other professional backgrounds (4.4%). The mean age of the assessors was 42.8 ± 11.8 years and 92.9% were women. These items were not included in the 2013 survey.

The questionnaire used consisted of the Multi-Dimensional Dementia Assessment Scale (MDDAS) [14, 15] which includes items covering activities in daily living (ADL), behavioral and psychological symptoms, and cognitive function. The subscale for assessing cognitive function was developed by Gottfries and Gottfries [15, 16]. Additional questions concerning pain and a registration of current drug prescription were included in the survey.

An ADL score was calculated based on the resident’s ability to cope with hygiene, dressing, eating, bladder and bowel control. All ADL items scored 1–5, apart from bladder control, which scored 1–4. The ADL score varies from 4 to 24, where a higher number represents greater ADL independence.

Cognitive impairment was measured using a scale developed by Gottfries and Gottfries [16]. The scale comprises 27 items measuring a person’s level of cognitive function. A score of less than 24 points indicates cognitive impairment, correlating with a sensitivity of 90% and specificity of 91% [14] to the usual 24/30 Mini-Mental State Examination (MMSE) [17] cut-off.

Pain was estimated according to answers of “Yes,” “No,” or “Do not know” to the following questions: Is the resident in pain? Does the resident feel pain during activity? Does the resident have pain when at rest? Does the resident receive treatment with drugs for his/her pain? Does the resident receive treatment other than with drugs for his/her pain? Does the treatment help? For the question “Is the resident in pain,” the correlation between proxy raters’ answers and self-reports were compared among 86 individuals aged 85 years or older (mean age 92.9 ± 4.6 years, 71 [82.6%] women): For individuals without dementia (MMSE 22.6 ± 3.4), the answers were in accordance for 16/17 (94.1%) individuals and for individuals with dementia (MMSE 12.0 ± 6.2) answers were in agreement for 48/69 (69.6%); correlations were 0.88 and 0.41 respectively [18].

A registered nurse filled in the data concerning prescribed medication from the resident’s prescription record. Drugs were coded, by the researchers, into different groups according to the WHO Anatomical Therapeutical Chemical classification index. The dosage was not coded. Analgesics were grouped into following categories: Analgesic (N02 + M01A), with subgroup opioid analgesic (N02A); paracetamol (N02B E); and non-steroidal anti-inflammatory drugs (NSAID) (M01A). Tramadol (N02AX02) was included in the N02A group but was also analyzed separately. Glucosamine (M01AX05) was excluded from the NSAID (M01A) category. Migraine medications (N02C) were excluded from this study.

The Regional Ethical Review Board in Umeå approved the study (registration number 07-028M and 2012-646-31M).

### Statistics

Comparisons were made using the Chi-square test and *t* test when appropriate. A *p* value < 0.05 was considered statistically significant. Analyses were performed using the Statistical Package for Social Science, SPSS version 22.

## Results

The basic characteristics of the samples are presented in Table 1. There was a difference in the residents’ ability to walk independently and their ability to make themselves understood, but there were no other differences in any of the investigated characteristics between 2007 and 2013.

The prescribed analgesic medication is presented in Table 2. Analgesic treatment had become more common in 2013 compared to 2007 (66.5% compared to 62.8%, *p* = 0.010). The use of opioids had increased significantly from 19.6 to 25.6% (*p* < 0.001), while the prescription of paracetamol and NSAID had not changed significantly. The use of tramadol had significantly decreased from 6.5 to 0.9% (*p* < 0.001) between 2007 and 2013.

**Table 2:** Regularly prescribed medication (ATC-code)

	2007	2013	*p* value
Analgesics (N02 + M01A) *n* (%)	1768 (62.8)	1255 (66.5)	0.010
Opioid analgesics (N02A) *n* (%)	551 (19.6)	483 (25.6)	< 0.001
Paracetamol (N02B E) *n* (%)	1663 (59.1)	1099 (58.2)	0.559
NSAID (M01A) *n* (%)	133 (4.7)	67 (3.6)	0.050
Tramadol (N02AX02) *n* (%)	183 (6.5)	17 (0.9)	< 0.001

*NSAID* non-steroidal anti-inflammatory drugs

The staff assessed 1565 residents in 2007 (63.4%) and 1183 in 2013 (62.3%) as experiencing pain (*p* = 0.499). Table 3 presents separately the proportions among women and men, and among individuals with and without cognitive impairment, respectively, in the two years.

**Table 3:** Prevalence of pain in different groups, as assessed by care staff

	2007	2013	*p* value
Individuals assessed as having pain, total, *n* (%)	1565/2468 (63.4)	1183/1899 (62.3)	0.499
Among women *n* (%)	1115/1701 (65.5)	865/1292 (67.0)	0.422
Among men *n* (%)	450/767 (58.7)	318/607 (52.4)	0.020
Among individuals with cognitive impairment, *n* (%)	1083/1733 (62.5)	851/1329 (64.0)	0.381
Among individuals without cognitive impairment, *n* (%)	456/699 (65.2)	316/541 (58.4)	0.014

Of the women, 64.5% in 2007 and 68.8% in 2013 were prescribed analgesics (*p* = 0.012), and among men the figures were 59.2% in 2007 and 61.6% in 2013 (*p* = 0.362). Among residents with cognitive impairment, 65.3% in 2007 and 66.9% in 2013 were prescribed analgesics (*p* = 0.316), and among residents who were cognitively intact, 56.6% were prescribed analgesics in 2007 and 65.6% in 2013 (*p* = 0.001).

Of those residents who were judged to be in pain, 1249 (79.8%) in 2007 and 892 (83.2%) in 2013 were prescribed at least one regular analgesic drug, hence 316 (20.2%) in 2007 and 180 (16.8%) in 2013 were untreated (*p* = 0.028). Table 4 presents separately the proportions among women and men, and among individuals with and without cognitive impairment, respectively, in the two years. In 2007, 16.7% of the residents with cognitive impairment had untreated pain compared to 28.5% of those who were cognitively intact (*p* < 0.001). In 2013, there was no significant difference regarding untreated pain between people with and without cognitive impairment (15.8% compared to 20.1%, *p* = 0.093). There was no significant gender difference in either of the years (data not shown).

**Table 4:** Proportion with at least one regularly prescribed analgesic medication, among individuals assessed as having pain

	2007 *n* = 1565	2013 *n* = 1183	*p* value
Individuals with regular analgesic treatment *n* (%)	1249/1565 (79.8)	892/1072 (83.2)	0.028
Among women *n* (%)	894/1115 (80.2)	652/792 (82.3)	0.239
Among men *n* (%)	355/450 (78.9)	240/280 (85.7)	0.021
Among individuals with cognitive impairment, *n* (%)	902/1083 (83.3)	652/774 (84.2)	0.585
Among individuals without cognitive impairment, *n* (%)	326/456 (71.5)	226/283 (79.9)	0.011

The staff member judged that 95.2% in 2007 and 85.1% in 2013 (*p* < 0.001) received treatment with medication for their pain. The staff member estimated that the treatment helped in 82.9% of the cases in 2007 and in 77.5% of the cases in 2013 (*p* = 0.002). Among those who were judged to be in pain but were not prescribed analgesics, the staff incorrectly answered that the resident was prescribed analgesics in 232 out of 316 (73.4%) cases in 2007 and in 135 out of 180 (75.0%) cases in 2013 (*p* = 0.699).

## Discussion

In line with previous research [19, 20], this study found that pain is a common condition among older people living in nursing homes. Almost one in five was untreated despite assessed as experiencing pain and among those with pain and no pharmacological treatment, the staff answered incorrectly that they received treatment in a large proportion of the cases. Further, the prescription of opioids became significantly more common while the use of tramadol decreased significantly from 2007 to 2013.

The use of opioid analgesics increased significantly between 2007 and 2013. The prevalence of NSAID was 3.6% in 2013 compared to 4.7% in 2007, a near significant decrease (*p* = 0.0502). Together, this may reflect current recommendations for the treatment of the pain in fragile elderly people [11]. Limiting the prescription of NSAID and considering instead the use of opioids is recommended, as opioids are well-known drugs that have been used for pain for many years. If opioid dosage is individualized and treatment is carefully monitored, with laxative therapy prescribed throughout, they may be associated with less risk and better analgesic effect than NSAIDs in older people. For old people, NSAIDs are potentially life-threatening drugs that have been implicated in 23.5% of hospital admissions in older people due to adverse drug reactions [11]. However, there is still only limited evidence available regarding the value of opioids [21] and it is known that opioids have side effects such as constipation [22], mental clouding [23], urinary retention [24], and can cause opioid induced hyperalgesia [25].

The most commonly used analgesic drug in 2007 and 2013 was paracetamol. Almost six out of ten were regularly prescribed paracetamol for pain treatment. These figures are in line with results from other studies [26]. There is evidence to suggest that paracetamol should be considered the “first-line treatment for both acute and persistent pain” as it has been shown to be both effective and safe as long as the maximum daily dose is not exceeded [2, 21].

The use of tramadol decreased significantly between 2007 and 2013 (6.7 to 0.9%). One possible explanation for this is that physicians have become more aware of the side-effects of tramadol among the elderly, e.g., confusion, dizziness, and reduction of the seizure threshold [27]. In addition, the National Board of Health and Welfare has published guidelines for drugs unsuitable for the elderly (including tramadol), and this has probably had an impact on prescribed medications. Another contributor to the reduced use of tramadol between 2007 and 2013 may be the regular drug utilization review that was introduced in 2012 in the county of Västerbotten. This was introduced against the background of a new regulation from The National Board of Health and Welfare stipulating that drug utilization reviews should be offered at least once a year to patients 75 years of age or older who are prescribed at least five drugs [28, 29].

The present study indicates that many people are not prescribed analgesic treatment despite being in pain. The proportion of residents with pain but not prescribed any analgesic treatment was less compared to the figures others have reported [13, 26, 30, 31] but still indicates an area for possible improvement.

The present study showed that the assessors, most often nursing assistants and licensed practical nurses, overestimated the number of residents with pain who were actually treated pharmacologically for their pain. This knowledge gap is in line with a study by Lövheim et al. [13], thus no improvement in this situation can be seen from 2000 to 2013. Lövheim et al. suggested that better communication between the various professional categories involved in nursing home care might improve the treatment of pain. Education of nursing home staff concerning the identification and management of painful conditions could also possibly help improve on this matter.

Dementia itself may be an obstacle in the management of pain [32]. Older adults in general have more difficulty communicating their pain [33] and this is exacerbated by the communication difficulties that are experienced by the majority of people with dementia, particularly in the later stages when language and cognition can be impaired [6]. It has been reported that there is a greater risk of under treatment of pain in people with dementia [33]. However, in this study, there was a greater use of analgesic drugs among people with cognitive impairment compared with residents who were cognitively intact. This is in line with previous studies undertaken in Scandinavia [21].

The strengths of the present study include the large number of participants and the unselected sample of people living in nursing homes. Drug registration was of a high quality. This study also, however, has some limitations. Firstly, the pain assessment used in this study is not a validated assessment, which would have been desirable. Also, the assessment was answered not by the residents themselves but by a staff member. Even if it was done by the staff member who knew the resident best (i.e., the resident’s key-worker), it is still a proxy estimation and a possible source of error as compared to self-reported symptoms. On the other hand, not all residents with cognitive impairment might be able to give valid estimations themselves, and low agreement between proxy-rated and self-rated symptoms could also stem from difficulties to remember and properly express pain, as well as from difficulties for the proxy rater to interpret symptoms of pain among individuals with severe cognitive impairment. A single question about pain might also be less sensitive as compared to questions regarding specific types of pain, and do not allow any grading of the intensity and frequency of pain. These limitations must be taken into account when interpreting the results. Nonetheless, as the same questions were administered both years, comparisons between the years might still be valid. Another limitation was a greater proportion of missing values in 2013 compared to 2007. Finally, this study was a one-week cross-sectional study; the data are point estimates and do not indicate causality.

## Conclusion

In conclusion, there has been a change in pharmacological analgesic treatment between 2007 and 2013 with less prescription of tramadol and a greater proportion using opioids. However, undertreatment of pain still occurs and staff members, who were supposed to know the residents best, believed in many cases that the residents were prescribed analgesic treatment despite the fact that they were not.
